# Ontario Veterinary Medical Association

**Published:** 1892-02

**Authors:** C. H. Sweetapple

**Affiliations:** Sec’y and Registrar Ont. Vet’y Association


*Ontario Veterinary Association.*—The annual meeting of the Ontario Veterinary Association was held in the Ontario Veterinary College, in Toronto, on December the 23d, 1891. The President, Mr. Gibb, of St. Mary's, Ont., in his opening addresses, gave some good advice. He strongly advocated the advantages of periodical meetings of the profession for purposes of discussion and mutual improvement.

Messrs. Robson Kidd and Sisson were duly proposed and elected members of the Association. On motion of Mr. Loyd, seconded by Prof. Smith, Principal of Ontario Veterinary College, Prof. Law, of Cornell, who was present, was unanimously elected an honorary member.

Mr. John Wende read an interesting paper on some cases of accidental poisoning of horses by castor oil bean mixed in the food, which had occurred in the course of his practice.

Mr. Cowan gave an address on Hog Cholera, of which he has had considerable experience; he mentioned the difficulties frequently occurring in diagnosis without a post-mortem examination. The temperature and pulse for various reasons are unreliable guides, also the skin lesions; blue, red or purple patches may or may not be seen. He mentioned the hæmorrhagic lung and ulcerations of the intestines as diagnostic. The ulcerations usually occurring in the glands in the neighborhood of the ilio-coecal valve. Prof. Law also gave some very interesting information in relation to the disease. Mr. Mole spoke of the tenderness of the skin to the touch which he had observed in Ireland. Mr. Cowan had not noticed this condition in Canada. Prof. Law mentioned that symptom as a peculiarity of the disease in Great Britain.

A discussion on changing the date of the annual meeting then took place. It was ultimately decided that instead of doing so this year, a meeting of the Association should be held in June, 1892, in the city of London, Ont.

The following officers were duly elected for the ensuing year : Mr. Macarthur, President ; Mr. John Wende, 1st Vice-President ; Mr. W. H. Burns, 2nd Vice-President ; Mr. C. H. Sweetapple, Secretary ; Mr. W. Cowan, Treasurer. Messrs. Hand, W. J. Wilson, Steele, Gallanough, Hopkins, Quinn, Kidd and Wiadefield, Directors. Messrs. Elliott and O'Neil, Auditors. Messrs. Wilson and O'Nei:, delegates to Western Fair Association. Mr. W. Cowan, representative to Central Farmer's Institute. Messrs. Wende, Cowan and Gibb were ap pointed to read papers at the meeting to be held in London, in June.

C. H. SWEETAPPLE,
*Sec'y and Registrar Ont. Vet'y Association.*

